# The Effects of Exam-Induced Stress on EEG Profiles and Memory Scores

**DOI:** 10.3390/bs13050373

**Published:** 2023-05-02

**Authors:** Taylor Roy, Kevin S. Saroka, Victoria L. Hossack, Blake T. Dotta

**Affiliations:** Behavioural Neuroscience Program, School of Natural Sciences, Laurentian University, Sudbury, ON P3E 2C6, Canada

**Keywords:** EEG, exam stress, memory, parahippocampal gyrus, cortisol

## Abstract

Common stressors amongst postsecondary students are exam-induced anxiety and stress. The purpose of this study was to measure stress alterations in the student population around examinations and determine how they affect electroencephalogram (EEG) profiles and memory scores. Twenty university students were measured multiple times in the study. During each measurement, participants were administered a cortisol saliva test and an EEG. We hypothesized that cortisol levels, memory scores, and EEG profiles would all demonstrate changes near examinations. The brain regions of interest (ROIs) were the parahippocampal gyrus, the medial frontal gyrus, and the middle frontal gyrus. Results demonstrated that memory performance and parahippocampal activity were correlated, specifically in the 5–9 Hz frequency band. Correlations were also computed between cortisol levels, memory performance, and parahippocampal activity. The medial frontal gyrus also displayed changes in the mean (19–20 Hz) current source density (CSD) throughout the experiment. The middle frontal gyrus activation was highly variable during the different measurement time points. Essentially, when an individual’s memory scores were consistent between exam and nonexam trials, there was an increase in middle frontal gyrus activation during examination periods. Lastly, the right parahippocampal gyrus was found to be the most activated one day away from examination time. These results indicate that memory scores are related to cortisol levels and examination periods, but most importantly, there are overt and predictable alterations in student EEG profiles near examinations.

## 1. Introduction

Stressful life events can trigger a series of processes within the central nervous system that result in the fight-or-flight-response and cortisol secretion [1]. The increase in cortisol secretion is known to suppress the immune system, alter the catabolic state, and increase energy [2]. The number of hormones secreted during a stress response depends on the severity of the stressful experience; however, there is a consistent correlation between the activation of the catecholamines, cortisol, and stress [1]. Research has shown that stress experienced long before learning can impair memory formation, whereas stress experienced shortly before or after the new information is presented can enhance memory performance [3].

Electroencephalography (EEG) is a noninvasive method for measuring electrical activity in the cortex of the brain. Because of this capacity, the EEG has become a powerful tool to predict and assess comfort levels, epilepsy, and depression [4,5,6]. Additionally, previous studies have shown alterations in the frequency of EEG activity during stress [7,8,9]. In the week before examinations, medical students were found to have increases in the frequency of their beta activity when compared to their baseline EEGs [9]. Another study found that when students took an exam while under pressure, there were decreases in frontal theta and alpha activity [8]. Hewig et al. [7] found that increases in cortisol after waking were correlated with increased alpha activity in the parietal lobe 6 weeks before the students’ exam period started and increased alpha activity in the left parietal lobe at the end of their exam period.

Cortisol is a steroid hormone, more specifically, a glucocorticoid, produced by the zona fasciculate of the adrenal gland [2]. Individual differences can be found in all tasks; some individuals may produce high levels or low levels of cortisol, and others may not respond to stressors at all [10]. Cortisol is the main glucocorticoid hormone, secreted by the adrenal glands in response to stress [11]. Administration of cortisol has been found to increase right frontal alpha activity [12] and increase the correlation between delta and beta wave activity [13].

The aim of this study was to investigate whether an increase in cortisol levels was associated with impaired working-memory retrieval and recognition in students. This within-subject experiment measured the concentration of cortisol and electroencephalographic profiles at the beginning of the semester and again during an examination period. Our regions of interest (ROIs) were the parahippocampal gyrus, the medial frontal gyrus, and the middle frontal gyrus. 

## 2. Methods

### 2.1. Participants

Students were recruited from undergraduate classes at the beginning of the semester from Laurentian University. Participation was completely voluntary; however, students from psychology classes were incentivized with a bonus percentage that would count towards their final grade. The experiment was reviewed and approved by the Laurentian University Research Ethics Board (LUREB; File No. 6016071). The sample consisted of 19 students: 8 males and 11 females. The mean age for these students was 21 years (±3 years). 

### 2.2. Memory Test

The memory test consisted of participants viewing a collage of black-and-white images for 30 s. There was a total of 17 images of objects in the collage. The images were nondescript and consisted of a baseball bat, a fruit basket, a candy cane, a fork, etc. At the end of the experiment (20 min), the participants were asked how many of the images they were able to recall. The average score for the nonexam period was 74.7% accurately recalled, and the average score for the exam period was 63.8% recalled.

### 2.3. Salivary Cortisol

Participants were asked to provide a sample of saliva during both the nonexam period and the exam period. This consisted of them collecting approximately 1 mL of saliva in an Eppendorf tube. Since cortisol levels have been shown to fluctuate at different times of the day [11], all samples were taken during the afternoon. The saliva collected in this experiment was stored at –20 °C for 1 month before it was examined, which has shown not to deteriorate the cortisol concentration [14]. The enzyme-linked immunosorbent assay (ELISA) was used to analyze the concentration of cortisol found in the saliva of each participant. 

### 2.4. Electroencephalogram

#### 2.4.1. Data Collection

On two separate occasions that reflected the nonexam and exam periods, each participant (*N* = 20) was asked to sit and relax in a chair. An EEG (electroencephalography) cap (Electro-Cap International Inc., Eaton, OH, USA) containing 19 sensors was placed on the participant’s scalp, with linked earlobes as reference sites. The scalp electrical activity was fed into a Mitsar-201 EEG amplifier (Mitsar Co. Ltd., St. Petersburg, Russia) connected to a Lenovo laptop computer (Lenovo Group Ltd., Beijing, China) running Windows 7. Spatial application of the electrodes was in accordance with the 10–20 international standard of electrode placement. A sodium-based gel was applied to each electrode in order to maximize the conductivity. Each trial measured brain activity for approximately 10 min (5 min eyes closed and 5 min eyes open).

#### 2.4.2. EEG Signal Processing and Extraction

Data were recorded with WinEEG software (version 2.127.98, Mitsar Co. Ltd., St. Petersburg, Russia) at a sample rate of 250 Hz. We applied a high-pass filter of 1.5 Hz, a low-pass filter of 50 Hz, and a double-notch filter which attenuated 60 Hz and 120 Hz powerline artifacts; all of these filters were applied within WinEEG software. Artifact correction was also completed within WinEEG, wherein indications of heart and eyeblink artifacts were removed via visual inspection. The data pertaining to 6 participants were removed, either because they did not return for their second EEG measurement or due to EEG recordings that were not viable after artifact correction. Therefore, the sample size available for the rest of the processing stage was *N* = 14.

Filtered and artifact-corrected, eyes-closed data collected during the time of each measurement (pre-exam and exam periods) were exported separately for each participant into an ASCII text document for further processing. Each eyes-closed recording was then segmented into thirty 5 s segments for later averaging; therefore, for each participant, there were a total of *N* = 30 pre-exam, eyes-closed segments and *N* = 30 exam-period segments.

**Figure 1:**
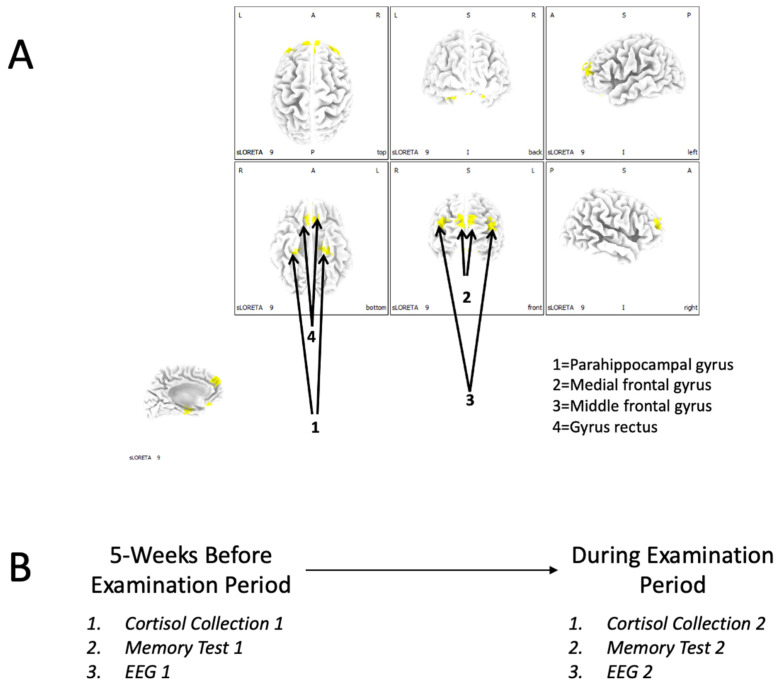
(**A**) The regions of interest (ROIs) that were adopted for analysis in this study. (**B**) Overall procedures employed to discern differences in neurochemistry, memory performance, and neurophysiology (EEG) parameters between the pre-examination and examination periods.

sLORETA (version 20221229, The KEY Institute for Brain-Mind Research, Zurich, Switzerland) is a collection of independent modules that can be used to conduct source localization of brain functions [15]. One of the features of this program is the derivation of current source densities (uA/mm^2^) for regions of interest (ROIs). Therefore, we conducted an ROI analysis to determine if there were any brain/behaviour relationships with regards to performance on the memory test, cortisol levels, and indications of brain functions in 4 regions of interest (MNI coordinates in parentheses): (1) parahippocampal gyrus (left: X = −27, Y = −17, Z = −20; right: X = 30, Y = −17, Z = −18), (2) the medial frontal gyrus (left: X = −4, Y = 58, Z = 23; right: X = 4, Y = 58, Z = 23), (3) the middle frontal gyrus (left: X = −38, Y = 55, Z = 23; right: X = 38, Y = 55, Z = 23), and (4) the gyrus rectus (left: X = −4, Y = 30, Z = −24; right X = 4, Y = 30, Z = −24). A text file specifying these ROIs was imported into “*ROI Maker 1*” (all voxels within 10 mm radius). These ROI data are depicted in Figure 1A.

Next, we imported all eyes-closed ASCII segments into the “*EEGs to cross-spectrum*” function. To examine brain activity in more detail at a finer frequency resolution, these cross-spectrum files were computed using custom-defined 1 Hz bins between 0 and 45 Hz rather than adopting the classical EEG frequency bands. We reasoned that adoption of a discrete frequency analysis would allow us to examine effects that are often hidden when frequency bands, which reflect the sum of the spectral densities (or spectral power) of discrete frequencies, are employed; the rationale was also inspired by the work of Klimesch, who reported that the alpha frequency band could be subdivided into sub-bands that are related to aspects of memory performance and attention [16]. During the computation of cross-spectral files, we enabled the “*Each BIG file to 1 CRS*” function to compute aggregates of each of the thirty 5 s segments for each participant’s pre-exam and during-exam eyes-closed segments. These data were subsequently entered into the “*Cross-spectrum to sLOR*” and then into the “*sLOR-to-ROI*” function for the computation of current source densities within the 0–45 Hz discrete frequency bands for each of the 4 regions of interest (left and right, separately).

### 2.5. Procedure 

The procedure adopted a repeated-measures (intragroup) design. On two different occasions (5 weeks before a scheduled exam *and* during their examination period), each participant provided a cortisol sample, had their EEG recorded, and completed a memory test. The overall procedure is depicted in Figure 1B.

## 3. Results

On average, participants scored 75.7% on the memory task during the first trial (nonexam period) and 63.1% during the second trial (exam period). For those who completed both trials (*N* = 18), a paired-samples t-test indicated that there was a significant (*t* = 2.96, *p* < 0.05) decrease in the performance of the task when participants completed the test closer to their examination. 

**Figure 2:**
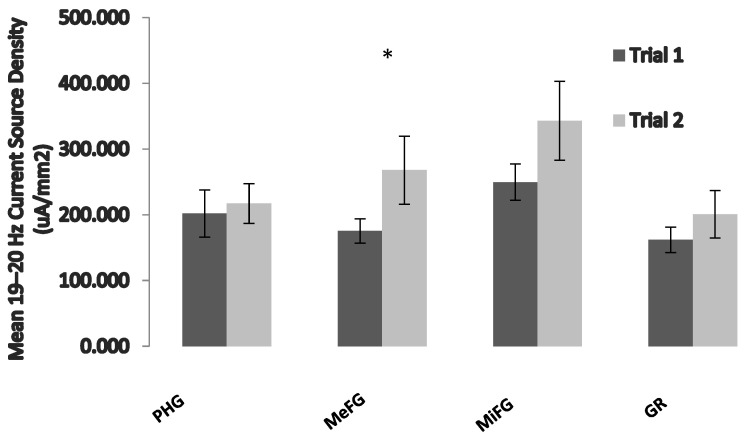
The effects of exam proximity on brain activity. Data were computed using the second trial only. Trial 1 refers to those who have already written their examination, and trial 2 refers to those who have not yet written it. PHG = parahippocampal gyrus, MeFG = medial frontal gyrus, MiFG = middle frontal gyrus, and GR = gyrus rectus. Vertical bars indicate standard errors of the mean (SEMs). * = *p* < 0.05.

In order to further identify the structures that may be involved in this decreased performance, a measure of examination proximity was computed by calculating the difference in the number of days between the EEG measurement and the student’s examination. Data were then recoded into a variable (exam time) consisting of two groups: already completed examination (*N* = 3) or to-complete examination (*N* = 11). Separate repeated-measures general linear model analyses were completed for each of the 1 Hz frequency bins, using one between-levels (exam time) and three within-level interactions (region, hemisphere, and trial) and using the current source densities of the ROIs. The results of the analysis indicated a significant region by trial interaction (*F = 3*.6, *p* < 0.05, partial eta2 = 0.23) only within the 19–20 Hz band. Post hoc paired t-tests revealed that the source of the interaction was an increase in the 19–20 Hz current source density (uA/mm2) within the bilateral medial frontal gyri around the time of the examination period (Figure 2). 

**Figure 3:**
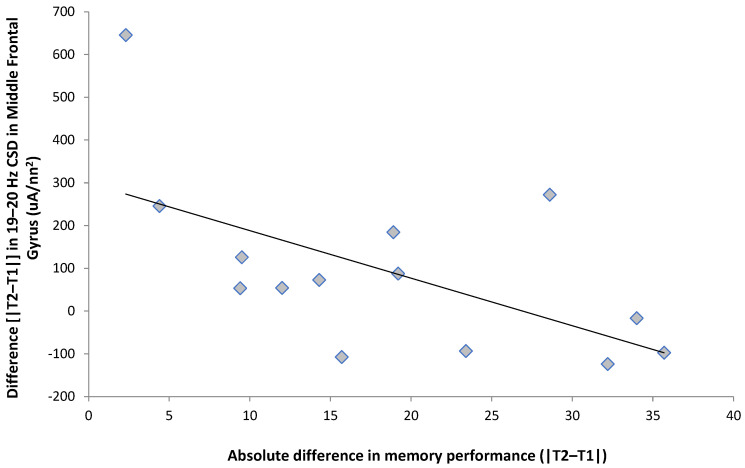
The scores on the test related to activity within the middle frontal gyrus. T1 refers to the first trial (nonexam period) and T2 refers to the second trial (exam period).

We then used this 19–20 Hz band as a proxy to conduct ancillary exploratory analyses, given that this was the only bin to demonstrate any statistically significant effects. To explore the effects within the 19–20 Hz band, we calculated a measure of the difference in performance in the memory task by taking the absolute difference between each participant’s scores during trial 1 and trial 2. We also calculated the difference (trial 2–trial 1) in the current source density for each of the four regions within the 19–20 Hz band; these differences were completed on the averaged left/right hemispheric activity, hence bilaterally representing the activity within each respective region. This score thus represents the magnitude of the difference in performance between the two trials. Nonparametric Spearman rank correlations were then completed between the magnitude of the participant’s performance difference between the two trials and the difference scores of brain activity between the two trials. This indicated that the magnitude of the difference in memory performance was significantly (Rho = −0.54, *p* < 0.05) and negatively correlated, with a 19–20 Hz current source density within the middle frontal gyrus (Figure 3).

**Figure 4:**
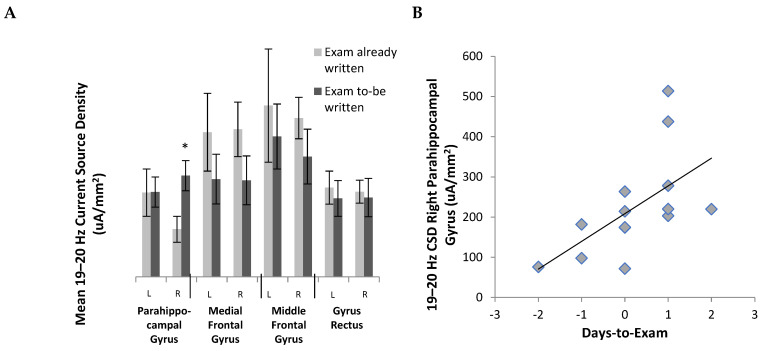
The parahippocampal gyrus is time-sensitive to examination period. (**A**) A significant activation in the right parahippocampal gyrus was observed between those who already wrote their exam and those who had upcoming exams, specifically in trial 2 only. * = *p* < 0.05. (**B**) Days-to-exam by right parahippocampal gyrus activity.

To discern if any of the regions selected for in the analysis were sensitive to the proximity examination within the 19–20 Hz band, we completed a series of Spearman rank correlations between the “time-to-examination” and current source densities within the various regions extracted during the sLORETA analysis. For this analysis, negative scores indicate that the exam was written X days before, 0 indicates that the participant was to write the examination on the day of trial 2, and positive values indicate how many days remain until the midterm examination. The results of the analysis indicated that the only significant relationship to emerge was a positive correlation between time-to-examination and CSD within the right parahippocampal gyrus (Figure 4B). The results of these analyses were confirmed using a repeated-measures GLM, with time as a between-levels interaction and region and hemisphere as within-level. The results indicated a significant three-way interaction (*F* = 3.85, *p* < 0.05, partial eta2 = 0.24) between testing day, hemisphere, and region. The results of the post hoc paired t-tests indicated that the right parahippocampal gyrus current source density was higher for individuals who had not yet completed their examinations (Figure 4A).

To determine if cortisol levels were affected by temporal proximity to a midterm examination, a paired t-test was completed. The results of the analysis indicated that there was no significant difference (*p* > 0.05) in cortisol levels, which suggests that cortisol was not altered during the midterm examination period. To investigate the relationships between memory, cortisol levels, and neuroelectric activity, all data were consolidated into one database consisting of each repeated measure as its own case. Because of the exploratory nature of this component of the analysis, we controlled for outliers by log transforming the ROI data; we have found this approach to be useful in exploratory analyses in minimizing spurious linear correlations driven by extreme cases. After the amalgamation of all available memory and cortisol data, there was a total of 27 valid cases. 

**Figure 5:**
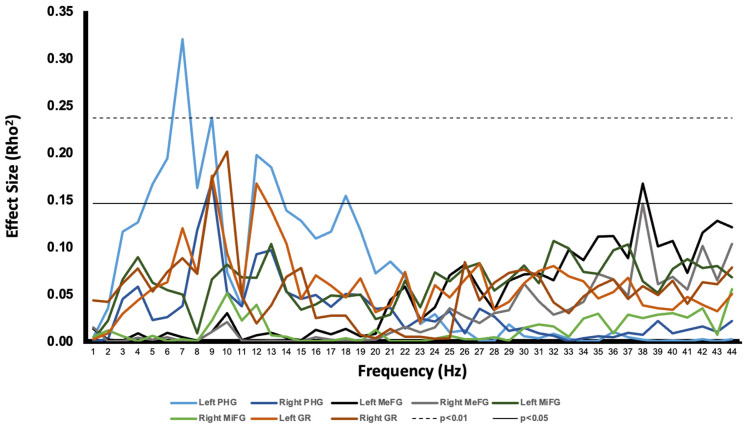
The relationship between memory performance and current source density of the ROIs (indicated by effect size), plotted as a function of frequency. In addition, the thresholds for significant correlations are indicated at *p* < 0.05 and *p* < 0.01.

Serial nonparametric correlational analyses were completed between memory scores and activity within each of the various regions extracted during the ROI analysis. The most consistent and strongest relationships found were between memory and low-frequency activity inferred from the left parahippocampal gyrus. Figure 5 displays a plot of the effect size for each bivariate correlation computed across each of the 45 frequency bins (0–45 Hz) for each region of interest. Because of the most prominent peaks within the 5–9 Hz band (Figure 5), it was assumed that parahippocampal activity within this band shared a common source of variance. Hence, a band between 5 and 9 Hz was constructed for the left parahippocampal region by averaging the spectral densities into one variable.

**Figure 6:**
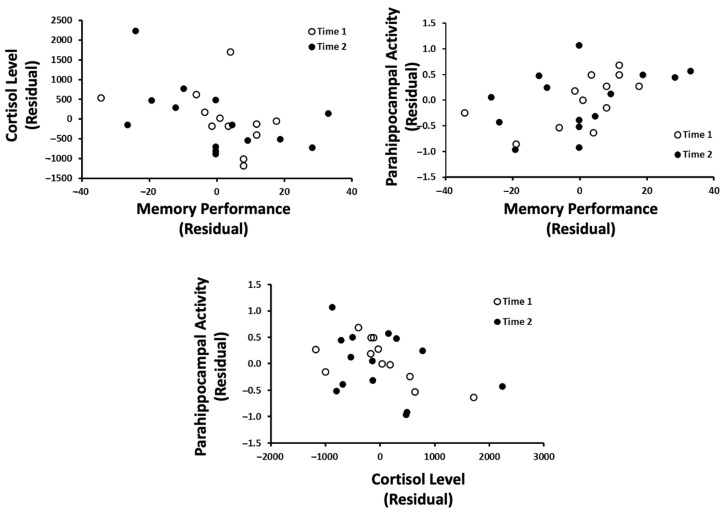
The relationship between cortisol levels, the parahippocampal gyrus, and memory performance. Time 1 refers to the first trial (nonexam period) and time 2 refers to the second trial (exam period).

Three multiple regressions were completed separately to obtain residual scores for memory, 5–9 Hz activity within the left parahippocampal region, and cortisol levels after controlling for the time that each measure was taken (long before exam = 1, shortly before exam = 2); this allowed us to examine the relationship between these three variables, irrespective of the participant’s proximity to exams. A series of nonparametric correlations were then completed (Figure 6). The results indicated that (1) cortisol levels were negatively correlated with memory performance (Rho = −0.48), (2) 5–9 Hz parahippocampal activity was positively correlated with memory performance (Rho = 0.54, *p* < 0.05), and (3) that 5–9 Hz parahippocampal activity was negatively correlated with cortisol levels. Stated alternatively, the results suggest that increasing cortisol levels are associated with lower performance on a memory task, which may or may not be dependent on 5–9 Hz parahippocampal activity in the left hemisphere.

## 4. Discussion

Optimized stress levels can increase an individual’s performance; however, extreme levels of stress can deteriorate it. The main goal of this research was to demonstrate a direct relationship between poor test scores, stress, and specific brain areas. Here, we demonstrated a relationship between memory performance, activity in the parahippocampal gyrus, and cortisol levels. Furthermore, we found structures, including the parahippocampal gyrus, the medial frontal gyrus, and the middle frontal gyrus, which were significantly influenced by the presence of an exam or the proximity of an exam.

Memory processing is a main function of the frontal lobe. In this experiment, a specific interest was found in the middle frontal gyrus—an area important for working memory [17]. When the process fails, working memory can be impaired, including not only the storage of information but also the simultaneous retrieval and processing of information in a capacity-limited store or computational workspace [18]. Those who had a greater difference in memory performance between the first and second trials displayed less brain activity in the middle frontal gyrus. This suggests that, when stressed, in order to perform as well as when one is relaxed, the brain, specifically the middle frontal gyrus, produces heighted activity to maintain those same results. Due to its role in working memory, these results are sensible.

Although there was no significant interaction between cortisol levels and proximity to midterm examination, a relationship between memory, cortisol levels, and neuroelectric activity was found. Data obtained showed a positive correlation between the parahippocampal gyrus and memory performance, demonstrating that when activity in the parahippocampal gyrus increased, a concurrent increase in memory scores was noted. Corroboratively, the literature indicates that it has many connections with brain regions that are known to be essential in memory processes (e.g., the hippocampus); thus, one of its main roles is memory formation [19]. This being said, it is not necessarily important for recollecting past memories, but it is important for new learning [20].

Predictably, negative correlations between cortisol levels and the parahippocampal gyrus activity, as well as between cortisol and memory performance, were observed. Stated another way, when cortisol levels were high, memory scores and parahippocampal gyrus activity were low. These results are consistent with the data found in the literature. Memory consolidation initially depends on the hippocampus, but the storage, retrieval, and more permanent memory development are distributed in regions of the neocortex, which is in part found in the parahippocampal gyrus [20]. Thus, the parahippocampal gyrus has a very important role in memory retrieval, and cortisol is discharged by a stress response. When cortisol is released, it disrupts the structures and impairs their capability to retrieve the information [10].

Furthermore, the parahippocampal gyrus, specifically the right hemisphere, had a higher current source density in individuals who had not yet written their exams compared to those who did. This brain region encompasses the limbic cortex, which is part of the limbic system [21]. The limbic system plays a vital role in behaviour and is activated during a stress response [21]. Our results solidify the information found in the literature since those who have not yet written their exams are expected to be more stressed and anxious than individuals who have already completed their examination period. Once a stressful situation is complete (in this case an exam), individuals seemed to be more relaxed and their parahippocampal gyrus activity was lower.

In summary, the results support the involvement of the parahippocampal gyrus in stress and confirms that all three regions of interest (the parahippocampal gyrus, the middle gyrus, and the medial gyrus) have roles in memory processing. All three regions are also correlated with memory performance and exam proximity. Lastly, a further outlook on the biological aspects demonstrated that the individuals were indeed stressed when memory performance was lower. These findings provide further support for the role of these brain regions. They have also given evidence that the stress response (the release of cortisol) does have a negative effect on memory performance. With this, we can conclude that examination stress can have a negative effect on examination results as it disrupts brain areas responsible for the retrieval of memory.

## 5. Conclusions

The data presented demonstrate the strong relationship between cortisol levels, memory performance, and EEG profiles. Brain regions involved in memory were predictably related to both cortisol levels and performance. Stress management can be an invaluable tool, identifying—through EEG measurement—when to administer examinations, which could increase performance and more accurately measure a student’s aptitude. More testing is clearly required, but these preliminary data are very revealing. Since we can predict which regions will show the greatest changes during increases in stress, and these same regions are associated with performance and memory, the optimization of testing times could increase the validity of examinations and be less taxing for students.

## Data Availability

Raw data pertaining to this study can be made available upon request.
